# Intentional Negotiation and Filling of Accessory Canals: A Case Series With Three‐Dimensional Segmentation and Volumetric Healing Assessment

**DOI:** 10.1111/iej.70079

**Published:** 2025-12-04

**Authors:** Lucas Pinto Carpena, Gabriel Lima Braz, Henrique Timm Vieira, Nadia de Souza Ferreira

**Affiliations:** ^1^ Dental School Federal University of Pelotas—UFPel Pelotas Rio Grande do Sul Brazil

**Keywords:** accessory canal, cone beam computed tomography, root canal anatomy, root canal treatment

## Abstract

**Introduction:**

Accessory canals (ACs) are anatomical features that may harbour residual infected tissue and contribute to persistent periradicular inflammation. Although filling of these structures is occasionally achieved passively, their intentional mechanical negotiation remains rarely documented in clinical practice. This case series aims to describe the clinical approach, outcomes and imaging strategies used in the intentional debridement and filling of ACs.

**Methods:**

Six teeth with identifiable ACs on cone‐beam computed tomography (CBCT), in association with bone resorption, were included. All cases underwent chemomechanical preparation using rotary instrumentation with 2% chlorhexidine gel, active saline irrigation and final EDTA activation with passive ultrasonic irrigation. Intentional mechanical debridement of ACs was performed using small‐diameter hand files. All fillings were performed with gutta‐percha and AH Plus Jet sealer. Follow‐up was performed using CBCT and periapical radiographs after a mean period of 10.16 months. Three‐dimensional segmentation was used to aid localization and planning.

**Results:**

All ACs were successfully negotiated and filled with endodontic sealer. Tomographic analysis confirmed evidence of partial or complete periradicular healing in all six cases. No patients presented with clinical symptoms at follow‐up.

**Conclusion:**

This case series presents successful intentional instrumentation and obturation of ACs, supported by CBCT‐based planning and follow‐up. Although limited by the lack of a control group, the findings highlight the potential role of CBCT imaging and segmentation in identifying and accessing complex anatomy. Further prospective studies are needed to determine the impact of this approach on long‐term clinical outcomes.

## 1 Introduction

Managing the internal morphology of the root canal system is a demanding task that frequently presents challenges, even for experienced endodontists. Among the anatomical complexities encountered, accessory canals (ACs), a branch of the main root canal that can communicate with the external surface of the root, pose difficulties during root canal treatment (American Association of Endodontists 2020; Ahmed et al. 2018). These structures have been reported to harbour necrotic and infected tissue following root canal treatment, potentially contributing to persistent apical periodontitis and clinical failure, although the extent of their role in treatment outcomes remains unclear due to the lack of robust evidence (Ricucci and Siqueira Jr. 2010).

Persistent bacterial infection within the root canal system remains the main cause of endodontic failure (Siqueira 2001). Based on this, it seems logical to pursue the instrumentation of ACs when present. However, this rationale encounters several limitations in clinical practice. First, ACs are often undetectable in routine periapical radiographs and are highly difficult to visualise during treatment (Strobel et al. 2017). Second, their small diameter greatly restricts the possibility of effective instrumentation, making mechanical debridement a more feasible approach than active shaping (Dammaschke et al. 2004). Third, ACs can exhibit severe curvatures relative to the main canal, which further hinders negotiation and instrumentation (Parirokh and Hatami 2022).

These morphological and technical challenges make the intentional instrumentation and filling of ACs a highly complex procedure. As a result, clinical documentation in the literature is scarce, and existing publications are limited to a small number of reports with varying levels of detail and follow‐up (Parirokh and Hatami 2022; Jiménez‐Rojas et al. 2024). In this context, the present study aims to expand the current body of knowledge by presenting a series of six well‐documented cases of intentional mechanical negotiation and filling of ACs, with cone‐beam computed tomography (CBCT) follow‐ups. By offering detailed documentation of clinical strategies and outcomes, this series provides relevant guidance for clinicians and contributes to the development of future research hypotheses in this underexplored area of endodontics.

## 2 Methodology

Informed consent was obtained from all individual participants included in this study. The study was approved by an institutional research ethics committee (protocol number 90404625.3.0000.5318). This report follows the 2020 Preferred Reporting Items for Case reports in Endodontics (PRICE) guidelines; a flowchart containing case details and an appropriate checklist were provided (Files S1 and S2) (Nagendrababu et al. 2020). The study also complies with the principles outlined in the Declaration of Helsinki.

All patients treated in a private endodontic practice during 2024 underwent CBCT imaging as part of the diagnostic workflow. All scans were systematically reviewed, and ACs were recorded whenever clearly identifiable. The present case series includes all consecutive cases in which ACs were visualised on CBCT and were associated with periradicular bone resorption, and these were subsequently subjected to intentional mechanical negotiation. The cases were performed by the same experienced endodontist (L.P.C.); all procedures were performed using a dental operating microscope (OPMI Pico, Zeiss, Oberkochen, Germany). For recording, an EOS Rebel T7i Camera (Canon, Tokyo, Japan) was used, coupled to a 50T‐type beam splitter (Zeiss). Initial and follow‐up CBCT scans were performed in all cases using the same high‐resolution tomograph Eagle Edge 0.2 FS (Dabi Atlante, Ribeirão Preto, São Paulo, Brazil) with a reduced field of view (FOV) (5 × 5 cm) and a voxel size of 0.075 mm.

ACs were identified on CBCT volumes using the method described by Bueno et al. (2021) and implemented in the EvolDX software. Coronal, sagittal and axial slices of ≤ 0.1 mm thickness were navigated with multiplanar reconstruction (MPR) adjustments to align with suspected hypodense tracts. ACs were confirmed based on the characteristic “line–line–dot” pattern observed across the three orthogonal planes, allowing differentiation from root fracture lines and subsequent visualisation in volumetric rendering. Metallic artefacts were minimised using the blooming artefact reduction (BAR) algorithm available in EvolDX (Estrela et al. 2025). The algorithm automatically adjusts parameters such as brightness and contrast to reduce hyperdense halos from metallic materials, improving visualisation of root canal outlines and periradicular bone contours. Corrected images were visually verified to ensure accurate representation of surrounding structures.

Additionally, three‐dimensional segmentations were performed based on the initial and follow‐up CBCT scans. The DICOM files were imported into BlueSkyBio software (Blue Sky Bio LLC, Grayslake, IL, USA) to generate an STL file of the tooth using the automatic segmentation tool. For the segmentation of the pulp space and periradicular bone resorption, 3D Slicer software version 5.8.1 (The Slicer Community; www.slicer.org) was used, employing manual tools to generate STL volumes. In this step, the volumes of bone resorption were also assessed, allowing the calculation of the periradicular bone resorption volume reduction rate (%). This rate was determined using the formula: [(initial volume − follow‐up volume) / initial volume] × 100, providing a quantitative indicator of healing progression. All generated files were refined using Meshmixer software (Autodesk Inc., San Rafael, CA, USA). For the final evaluation of the segmentation accuracy, all files were imported into BlueSkyBio and visualised overlaid on the CBCT slices. These procedures were performed by a trained oral and maxillofacial radiologist.

## 3 Results

### 3.1 Standardisation of Procedures

**FIGURE 1:**
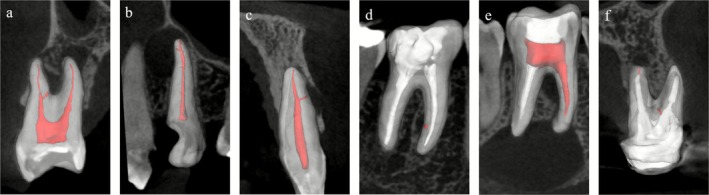
Three‐dimensional segmentation of the teeth and pulp spaces overlaid on the initial tomographic slices. (a) case 1; (b) case 2; (c) case 3; (d) case 4; (e) case 5; (f) case 6.

All six cases included in this series followed standardised clinical procedures. In all teeth, pulp necrosis was confirmed by a negative response to thermal (cold) testing. Periapical diagnosis was established based on a combination of clinical findings and imaging data. The presence of at least one accessory canal (AC) was confirmed preoperatively using CBCT (Figure 1). All treatments were performed under local anaesthesia and strict aseptic conditions, including rubber dam isolation and operative field decontamination.

**FIGURE 2:**
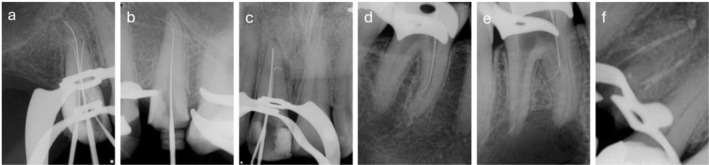
Periapical radiograph after mechanical access of the accessory canal. (a) case 1; (b) case 2; (c) case 3; (d) case 4; (e) case 5; (f) case 6.

Chemomechanical preparation of the main root canals was carried out using rotary instrumentation up to size 40.05 (Easy Bassi, Minas Gerais, Brazil). Working lengths for both the main canals and the ACs were determined electronically using a RomiApex A‐15 apex locator (Romidan LTD, Kiryat Ono, Israel), with the final length established at the “0.0” reading (Figure 2). Irrigation protocols were standardised across all cases, employing 2% chlorhexidine gel as a chemical adjunct and active irrigation with saline solution. Final irrigation was performed with 17% EDTA, activated using passive ultrasonic irrigation (PUI) in three 20‐s cycles per canal.

**FIGURE 3:**
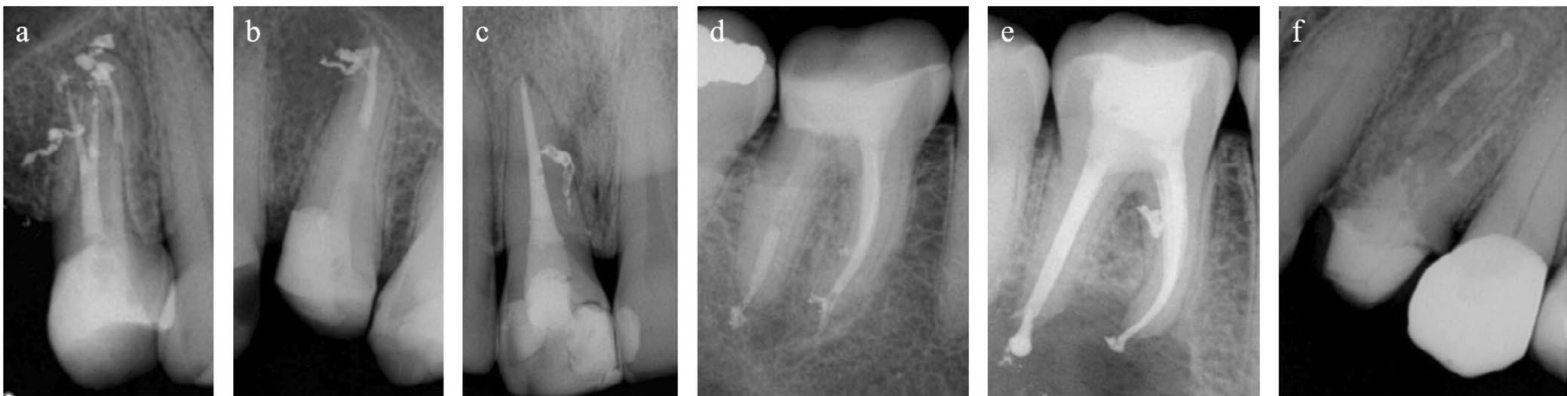
Final periapical radiograph after root canal system obturation. (a) case 1; (b) case 2; (c) case 3; (d) case 4; (e) case 5; (f) case 6.

**FIGURE 4:**
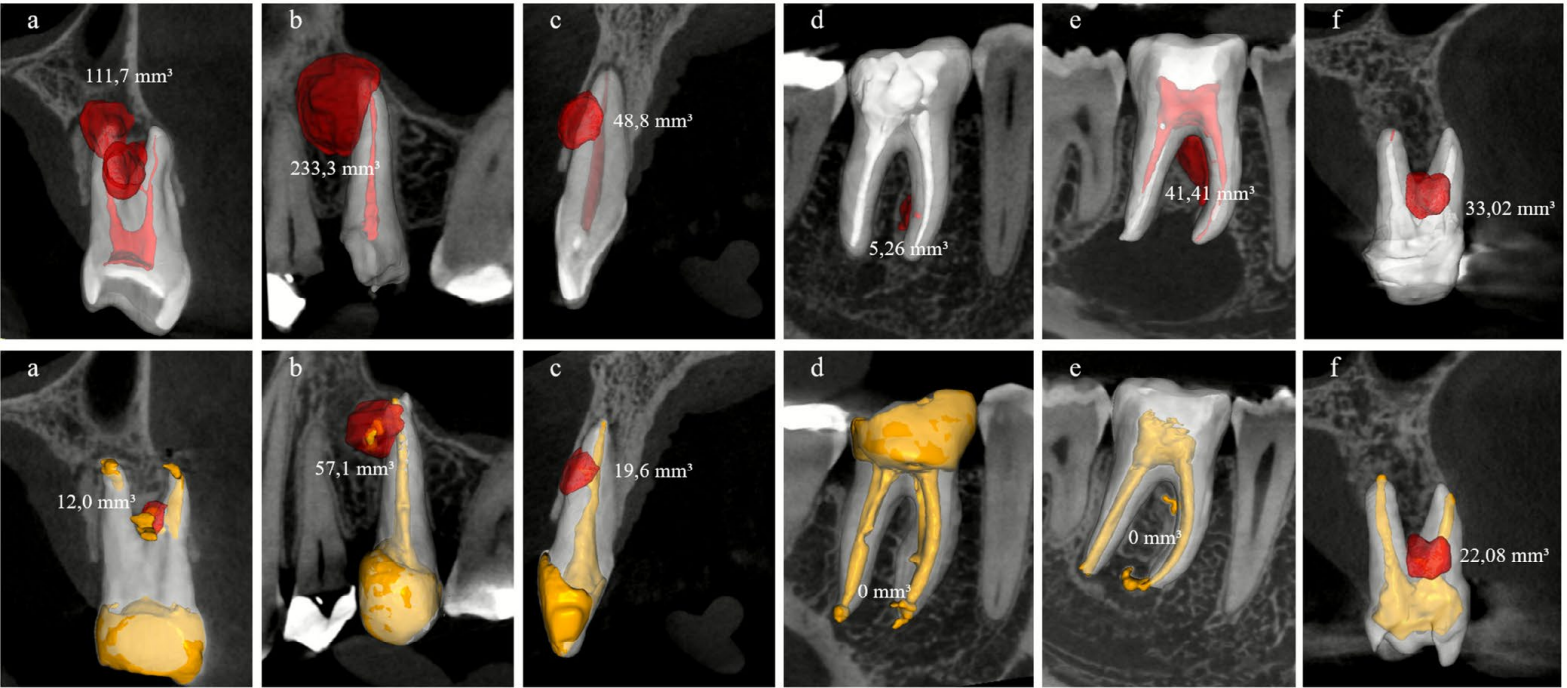
Tomographic follow‐up with three‐dimensional segmentation and volume of bone resorption associated with accessory canal. Initial tomography (red); Tomographic follow‐up (yellow); (a) case 1; (b) case 2; (c) case 3; (d) case 4; (e) case 5; (f) case 6.

All canals were obturated using gutta‐percha and AH Plus Jet sealer, following Schilder's vertical condensation technique and the teeth were immediately restored with composite resin (Figure 3). Radiographic and tomographic follow‐up was conducted between 6 and 14 months post‐treatment (mean: 10.16 months) (Figure 4). At follow‐up, all patients were asymptomatic. Table 1 summarises the demographic and clinical characteristics of the included cases.

**TABLE 1:** Clinical and demographic features of the case series.

Variable	Case 1	Case 2	Case 3	Case 4	Case 5	Case 6
Age	54	56	33	48	19	75
Sex	Female	Male	Female	Female	Female	Male
Tooth	15	25	11	46	46	24
AC classification^a^	^2^15 B^(M1)^ P	^1^25^(A1)^	^1^11^(A1)^	^2^46 M^(M1)^ D	^2^46 M^(C1)^ D	^2^24 B^(C1)^ P
AC angulation^b^	132°	107°	103°	92°	90°	127°
Periapical diagnosis	Symptomatic AP	Symptomatic AP	Symptomatic AP	Asymptomatic AP	Acute apical abcess	Chronic abcess
Intrumentation in the AC	K #10 K #15	K #10 K #15	K #10 K #15 K #20	K #10 K #15 K #20	K #10 K #15	K #10 K #15 K #20 Rotary 15.05 25.05
Intracanal dressing	No	No	No	No	Ca (OH)_2_ + 2% CHX (14 days)	No
Follow‐up	8 months	11 months	13 months	9 months	14 months	6 months
Volumetric bone resorption—initial value → follow‐up value (mm^3^)	111.7 → 12	233.3 → 57.1	48.8 → 19.6	5.26 → 0	41.41 → 0	33.02 → 22.08
Periradicular bone resorption volume reduction rate (%)	89.3%	75.5%	59.8%	100%	100%	33.11%

^a^ Classification according to Ahmed et al. (2018).

^b^ Degree of curvature in relation to the main canal; AC (Accessory canal); CBCT (Cone‐Beam Computed Tomography); AP (Apical Periodontitis); CHX (chlorhexidine).

### 3.2 AC Penetration Technique

CBCT scans were navigated in the e‐Vol DX software (CDT Software; Bauru, SP, Brazil), to identify the emergence point of the ACs, evaluate their angulation in relation to the main canal and measure their distance from clinically reproducible anatomical landmarks, such as cusp tips. These measurements guided the positioning of the instrument in the operative field. After complete preparation of the main canal, a size #10 K‐file was pre‐curved in its last millimetre using a college cotton plier, attempting to reproduce the degree of curvature suggested by the CBCT analysis. The rubber stop on the file was adjusted to the measured distance between the AC and the selected reference point (such as a cusp tip). The file was then introduced into the main canal with its curved tip oriented toward the root surface where the AC was located. Gentle watch‐winding and balanced‐force motions were employed to explore the canal pathway and verify patency. Once tactile sensation suggested penetration into the AC, the apex locator (RomiApex A‐15, Romidan LTD, Kiryat Ono, Israel) was connected to confirm canal entry and determine the working length, which was established at the “0.0” reading. Once negotiation was achieved, the glide path was confirmed with a #15 K‐file, and enlargement up to #20 was performed when possible. In highly curved ACs (> 120°), only patency and light debridement were attempted to avoid procedural errors.

**FIGURE 5:**
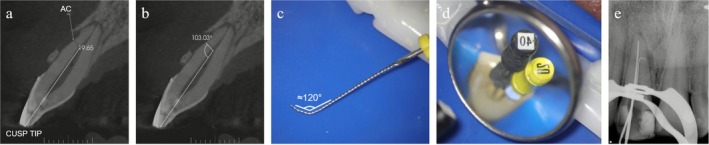
Accessory canal negotiation technique. (a) Measurement of the distance from the accessory canal to the incisal edge; (b) measurement of the angle formed between the accessory and main canal; (c) pre‐curved instrument; (d) instruments inserted into the main and accessory canal; (e) periapical radiograph after accessing the accessory canal.

In ACs located in the cervical third, with milder curvatures (< 120°) and a clear insertion pathway, mechanical instrumentation may be attempted in a comparatively safer manner using heat‐treated nickel–titanium mechanised files. The same pre‐curving technique applied to manual instruments can be used before activation, taking advantage of the alloy's thermal treatment and flexibility. Ideally, such instruments should combine high flexibility, resistance to cyclic fatigue and controlled shape memory—features that facilitate negotiation and enlargement while reducing the risk of file separation. Figure 5 demonstrates the planning and execution steps of this technique.

### 3.3 Case 1

A 54‐year‐old female patient was referred for root canal treatment due to pain on percussion. Tooth #15 presented with pulp necrosis and symptomatic apical periodontitis, as well as an AC located in the middle third of the palatal aspect of the buccal root canal (^2^15 B^(M1)^ P). The AC was intentionally debrided using size 10 and 15 K‐files (Dentsply, Ballaigues, Switzerland). Root canal filling was completed in a single visit. An 8‐month follow‐up demonstrated partial periradicular healing.

### 3.4 Case 2

A 56‐year‐old male patient was referred for root canal treatment due to pain on percussion. Tooth #25 presented with pulp necrosis and symptomatic apical periodontitis, along with an AC located in the apical third on the mesial aspect of the root canal (^1^25^(A1)^). During treatment, all existing restorative material was removed. The AC was intentionally debrided using size 10 and 15 K‐files (Dentsply, Ballaigues, Switzerland). Root canal filling was completed in a single visit. An 11‐month follow‐up revealed evidence of partial periradicular healing.

### 3.5 Case 3

A 33‐year‐old female patient was referred for root canal treatment due to pain on percussion. Tooth #11 presented with pulp necrosis and symptomatic apical periodontitis, along with an AC located in the apical third on the buccal aspect of the root canal (^1^11^(A1)^). The AC was intentionally debrided using size 10, 15 and 20 K‐files (Dentsply, Ballaigues, Switzerland). Root canal filling was completed in a single visit. A 13‐month follow‐up revealed evidence of partial periradicular healing.

### 3.6 Case 4

A 48‐year‐old female patient was referred for root canal retreatment due to pain on percussion. Tooth #46 had a previous root canal treatment and a metallic intracanal post in the distal canal. An AC was located in the middle third on the distal aspect of the mesiolingual root canal (^2^46 M^(M1)^ D). During the intervention, the metallic post was removed using ultrasonic tips (Helse Ultrasonic, São Paulo, Brazil). The AC was intentionally debrided using size 10, 15 and 20 K‐files (Dentsply, Ballaigues, Switzerland). Root canal filling was completed in a single visit. A fibre post was immediately cemented in the distal canal. A 9‐month follow‐up revealed evidence of complete periradicular healing.

### 3.7 Case 5

A 19‐year‐old female patient was referred for root canal retreatment due to pain on percussion. Tooth #46 had a previous root canal treatment, along with an AC located in the middle third on the distal aspect of the mesiolingual root canal (^2^46 M^(C1)^ D). An initial emergency appointment was performed, involving access through the existing restoration and chemomechanical preparation using a reciprocating R25 file (VDW, Munich, Germany) for removal of the previous obturation material and drainage of the intraosseous abscess that was causing severe pain. The tooth was medicated with 2% chlorhexidine gel and temporarily sealed.

In the second session, the main canals were prepared and the AC was intentionally debrided using size 10 and 15 K‐files (Dentsply, Ballaigues, Switzerland). The canals were then medicated with calcium hydroxide paste mixed with 2% chlorhexidine gel. During the third session, the main canals were re‐instrumented using size 40.05 files, and further debridement of the AC was performed. Root canal filling was completed in the third session (Dentsply, Ballaigues, Switzerland). 14‐month follow‐up demonstrated evidence of complete periradicular healing.

### 3.8 Case 6

A 75‐year‐old male patient was referred for root canal retreatment due to the presence of a chronic abscess, despite the absence of symptoms. Tooth #24 had a previous root canal treatment and presented with a cast metal post in both canals, covered by a ceramic crown. An AC was located in the cervical third on the palatal aspect of the buccal root canal (^2^24 B^(C1)^ P), which was considered the likely cause of the sinus tract.

During treatment, the ceramic crown and cast post were removed. Chemomechanical preparation was initiated using a reciprocating R25 file (VDW, Munich, Germany) to remove the previous filling material. The AC was intentionally debrided using size 10, 15 and 20 manual K‐files (Dentsply, Ballaigues, Switzerland), as well as 15.05 and 25.05 rotary files (Easy Bassi, Minas Gerais, Brazil). This active instrumentation was possible due to the coronal location of the AC and favourable angle of access with the rotary files. Root canal filling was completed in a single visit. Six‐month follow‐up demonstrated evidence of partial periradicular healing. The sinus tract was completely resolved at the time of follow‐up.

## 4 Discussion

The presence of ACs has long been recognised as a challenging anatomical feature in endodontics. Although traditionally considered less critical in treatment outcomes, some reports suggest that these structures may contribute to persistent periradicular inflammation and treatment failure due to the retention of infected tissue (Ricucci et al. 2013). This case series explores the intentional mechanical negotiation and filling of ACs in six cases, followed for a mean of 10.16 months with CBCT imaging, with favourable periradicular healing observed in all treated teeth.

Missed canals are a well‐established cause of post‐treatment apical periodontitis; however, this discussion is typically focused on main root canals (Costa et al. 2019). Currently, no clinical studies have analysed the association between detectable AC and the presence of periradicular bone resorption. In the present case series, all ACs were associated with periradicular bone resorption. Importantly, all included ACs were patent, which could hypothetically explain their potential role in maintaining periradicular resorption, as open canal pathways may facilitate the persistence of irritants or microbial activity beyond the main canal system. Notably, three of the included cases involved endodontic retreatment, and only one presented with a clearly unsatisfactory filling (> 3 mm short of the apex), supporting the hypothesis that untreated ACs may have contributed to the persistence of periradicular pathology.

Untouched areas within the root canal system are a frequent topic of discussion in contemporary endodontic practice, especially with the growing use of advanced imaging technologies (Siqueira Junior et al. 2018). It is well established that mechanical instrumentation alone is unable to contact all internal surfaces of the root canal system (Lopes et al. 2018). As a result, the chemical phase of treatment has gained increasing relevance. Several studies have demonstrated the significant impact of irrigant activation in reducing bacterial load. However, it is important to emphasise that adequate access cavity and mechanical instrumentation are essential to facilitate effective irrigant distribution (Brisson‐Suárez et al. 2024; Nogales et al. 2025; Vieira et al. 2020). This reinforces the potential benefits of mechanically penetrating ACs, not only to remove tissue but also to allow deeper irrigant reach. In most ACs, achieving proper shaping with larger taper instruments is impractical due to their extreme curvatures and limited diameter, which increase the risk of file separation even with heat‐treated alloys. Thus, small hand files remain the most reliable approach. However, under favourable anatomical conditions—such as coronal location, mild curvature and a straight insertion path—mechanised instrumentation may be considered to further enhance cleaning and irrigant exchange. In the present series, all cases underwent three 20‐s cycles of PUI with 17% EDTA during the final irrigation protocol. Although PUI improves penetration of irrigants, it may not always be sufficient to fully address ACs, particularly when tissue remnants are enmeshed within these structures (de Gregorio et al. 2010). In this context, mechanical negotiation should be viewed as a complementary approach to enhance irrigant effectiveness, rather than an alternative or competing strategy.

Filling of the ACs via sealer extrusion was achieved in all cases within this series. However, the common assumption that sealer extrusion alone substantially improves treatment success may be overly simplistic. Microbiological studies indicate that even when sealer is extruded into the AC, residual inflamed tissue can remain embedded within the canal alongside the filling material (Ricucci and Siqueira Jr. 2010). Although the clinical impact of mechanically addressing ACs remains uncertain, their debridement, when feasible, may help reduce residual infected or inflamed tissue, acknowledging that complete sterilisation of the root canal system is unattainable. In this context, alternative obturation approaches—such as carrier‐based systems or warm vertical compaction—may offer theoretical advantages for more controlled delivery of sealer. However, existing laboratory and clinical data are limited and inconsistent regarding whether these techniques reduce sealer extrusion into the periodontal space or translate into improved clinical outcomes. Therefore, while these methods warrant consideration, their potential benefits in the context of ACs remain hypothetical and should be evaluated in future comparative studies.

All cases in this series were performed under operative microscopy and guided by CBCT‐based planning. It is important to highlight that the successful negotiation of ACs relies heavily on the use of advanced technological resources and the clinician's experience. Modern CBCT software enables precise measurement of the AC's emergence point and its distance from reproducible anatomical references, which has the potential to enhance the predictability and accuracy of this procedure (Espinoza et al. 2024). As the global adoption of endodontic technologies continues to grow, the need for adequate training in CBCT interpretation becomes increasingly critical (Cheung et al. 2023). In this context, a close collaboration between endodontists and oral radiologists is essential, as effective communication can optimise the use of the acquired tomographic volume and improve clinical outcomes (Cheung et al. 2024). Although current guidelines do not routinely recommend CBCT for follow‐up, its use was considered appropriate in this study due to the limitations of periapical radiographs in detecting subtle periradicular changes, especially in posterior teeth. In such cases, ACs may produce vague or overlapping radiographic findings that suggest their presence yet fail to reveal a discernible canal lumen. CBCT imaging offers superior sensitivity in these scenarios and allows for more accurate visualisation of bone healing, particularly at the foraminal termination of ACs. As demonstrated by Brochado Martins et al. (2023), CBCT can reveal periradicular status changes not evident on conventional radiographs, supporting its value in selected follow‐up situations. The use of CBCT‐based segmentation also opens possibilities for education and pre‐clinical training (Cameron et al. 2023). Since the segmented volumes can be exported as STL files, they may be 3D printed to create pre‐clinical models of AC anatomy. Such models could allow clinicians to practice AC negotiation in a controlled environment, potentially, reducing risks when performing these procedures in clinical settings.

A previous micro‐CT study reported a mean diameter of 67 μm for accessory canals (Xu et al. 2016), suggesting that many may be near or below the detection threshold of high‐resolution CBCT scans (75 μm voxel in this study). While smaller voxel protocols could theoretically improve detection, they also increase radiation exposure, which may not be justified given the lack of evidence that visualising or treating such fine canals improves outcomes. Beyond diameter, the three‐dimensional configuration of ACs, particularly their degree of curvature in relation to the main root canal, has not been comprehensively evaluated in the endodontic literature. Although some reports describe ACs with sharp angulations, no quantitative data are currently available to inform clinicians about the typical or critical curvature thresholds that might compromise negotiation or instrumentation (Parirokh and Hatami 2022; Jiménez‐Rojas et al. 2024). This represents a relevant gap in knowledge, as the angle between the main canal and an AC may directly influence both the feasibility of mechanical negotiation and the risk of procedural complications, such as instrument fracture or canal transportation. In this case series, CBCT analysis revealed the presence of curvatures ranging from 90° to 132°, which made their negotiation particularly challenging. Future studies designed to evaluate the angulation and trajectory of ACs using CBCT may contribute significantly to treatment planning and case selection in AC negotiation.

The present case series has inherent limitations, including the small sample size, its single‐center design and the influence of operator experience, as all procedures were performed by a single highly experienced endodontist. This factor may limit the generalizability of the findings to less experienced practitioners. Additionally, the relatively short mean follow‐up period (10.16 months) restricts conclusions regarding the long‐term stability of the observed healing. Controlled clinical studies are therefore needed to determine whether intentional AC negotiation has any measurable impact on treatment outcomes beyond conventional therapy. Despite these limitations, this case series contributes to the growing body of evidence on the clinical management of ACs, providing the most thoroughly documented set of cases involving intentional instrumentation of these anatomical structures. Its strengths lie in the systematic use of CBCT for both preoperative identification and postoperative evaluation—including volumetric analysis of periradicular healing—as well as in the detailed step‐by‐step description of a complex and rarely reported technique. By integrating advanced imaging with clinical reproducibility, this study bridges the gap between technological capability and clinical application. Although the favourable outcomes observed cannot be directly attributed to AC instrumentation, the main contribution of this report lies in offering a reproducible description of the technique and generating hypotheses for future research.

## 5 Conclusion

This case series provides a detailed clinical and tomographic description of intentional instrumentation of ACs in six teeth, with all cases showing evidence of periaradicular healing after a mean follow‐up of 10.16 months. Advanced planning with CBCT imaging and operative microscopy was a key element in identifying and negotiating these anatomical structures. Although the outcomes were favourable, these findings must be interpreted with caution due to the inherent limitations of case‐based research. Nonetheless, the technical strategies and observations reported may help inform future studies and support the development of more predictable approaches to managing complex root canal anatomies.

## Author Contributions

All authors have contributed significantly to this paper (writing, editing and final review) and are in agreement with the final version of the manuscript. The conception and design of the study were conducted by Lucas Pinto Carpena and Nadia de Souza Ferreira. Data acquisition was performed by Lucas Pinto Carpena and Henrique Timm Vieira. Data analysis and interpretation were carried out by Lucas Pinto Carpena, Gabriel Lima Braz, Henrique Timm Vieira and Nadia de Souza Ferreira. The initial draft of the article was prepared by Lucas Pinto Carpena, Gabriel Lima Braz, Henrique Timm Vieira and Nadia de Souza Ferreira. All authors approved the final version of the manuscript.

## Funding

The authors have nothing to report.

## Disclosure

Relevant reporting guidelines paperwork: This manuscript was prepared following the 2020 Preferred Reporting Items for Case reports in Endodontics (PRICE) guidelines for case reports in Endodontics; a flowchart containing case details and an appropriate checklist were provided in Files S1 and S2.

## Ethics Statement

This study was conducted in accordance with the ethical standards of the institutional research committee and with the 1964 Helsinki declaration and its later amendments. Ethical approval was obtained from the Federal University of Pelotas ethics in research committee, under protocol number (90404625.3.0000.5318).

## Consent

Written informed consent was obtained from all individual participants included in the study.

## Conflicts of Interest

The authors declare no conflicts of interest.

## Supporting information


**File S1:** iej70079‐sup‐0001‐supinfo01.pdf.

**File S2:** iej70079‐sup‐0002‐supinfo02.pdf.

## Data Availability

The data that support the findings of this study are available on request from the corresponding author. The data are not publicly available due to privacy or ethical restrictions.
