# Supplementary material for: Intentional Negotiation and Filling of Accessory Canals: A Case Series With Three‐Dimensional Segmentation and Volumetric Healing Assessment
Source: Int Endod J. 2025 Dec 4;59(4):728–36. doi: 10.1111/iej.70079 (PMC12977934; doi:10.1111/iej.70079)
Supplement: Supplementary file 1 — File S1: iej70079‐sup‐0001‐supinfo01.pdf. [file IEJ-59-728-s002.pdf]

PRICE 2020 Flowchart

PATIENT DEMOGRAPHIC DETAILS
1. Female, 54 years-old 2. Male, 56 years-old 3. Female, 33 years-old 4. Female, 48 years-old 5. Female, 19 years-old 6. Male, 75 years-old

PATIENT CONCERNS/SYMPTOMS
1. Pain on percussion 2. Pain on percussion 3. Pain on percussion 4. Pain on percussion 5. Pain on percussion 6. Presence of a sinus tract

Informed consent was obtained from all patients

Patient's medical history was not contributory for diagnostics

PREVIOUS DENTAL HISTORY
Cases 4, 5 and 6 had undergone previous root canal treatment

CLINICAL FINDINGS
Case 6 presented a sinus tract

DIAGNOSTIC TESTS PERFORMED AND THEIR RESULTS
All patients were submited to thermic, percussion and palpation tests, as well as radiographic and tomographic examination. For all cases, thermic (cold) test was negative and percussion test was positive. Palpation test was positive only on case 5

DIFFERENTIAL DIAGNOSIS
A negative response to the cold test suggested necrosis of the pulp tissue, a positive response to the percussion test suggested a inflamattory condition of the apical periodontum

DEFINITIVE DIAGNOSIS
1. Symptomatic apical periodontitis 2. Symptomatic apical periodontitis 3. Symptomatic apical periodontitis 4. Asymptomatic apical periodontitis 5. Acute apical abcess 6. Chronic abcess

MANAGEMENT OPTIONS CONSIDERED
Cases 1-3: Non-surgical root canal treatment; Cases 4-6: Root canal retreatment

INFORMED, VALID CONSENT FOR TREATMENT
was obtained from all patients

TREATMENT/INTERVENTIONS PERFORMED (if any)
Cases 1-3: Non-surgical root canal treatment; Cases 4-6 : Root canal retreatment

FOLLOW-UP PERIOD(S): 1. 8 months; 2. 11 months; 3. 13 months; 4. 9 months; 5. 14 months; 6. 6 months.

FOLLOW-UP ASSESSMENT METHOD(S): Clinical examination, periapical radiography, CBCT (volume of bone resortion)

TREATMENT OUTCOME: All cases presented complete or partial reduction of the periapical lesion

PATIENT PERSPECTIVE: All patients were asymptomatic at follow-up

CONCLUSION(S): Mechanical access to accessory canals is a feasible technique, advanced CBCT planning may enhance it's reproductibility

This study recieved no funding

The authors declare no conflict of interest

***From: Nagendrababu V, Chong BS, McCabe P, Shah PK, Priya E, Jayaraman J, Pulikkotil SJ, Setzer FC, Sunde PT, Dummer PMH (2020) PRICE 2020 Guidelines for reporting case reports in Endodontics: A consensus-based development. *International Endodontic Journal* doi: 10.1111/iej.13285.**

For further details visit: http://pride-endodonticguidelines.org/price/
