# Supplementary material for: Intentional Negotiation and Filling of Accessory Canals: A Case Series With Three‐Dimensional Segmentation and Volumetric Healing Assessment
Source: Int Endod J. 2025 Dec 4;59(4):728–36. doi: 10.1111/iej.70079 (PMC12977934; doi:10.1111/iej.70079)
Supplement: Supplementary file 2 — File S2: iej70079‐sup‐0002‐supinfo02.pdf. [file IEJ-59-728-s001.pdf]

PRICE 2020

Checklist of items to be included when reporting case reports in Endodontics*

Section/Topic	Item number	Checklist Item	Reported on page number
**Title**	1a	The words "case report(s)" must be included in the title	1
	1b	The area of interest (e.g. anatomy, disease, treatment) must be included briefly in the title	1
**Keywords**	2a	At least two relevant keywords, preferably MeSH terms, related to the content of the case report must be included	1
**Abstract**	3a	The Introduction must contain information on how the report is novel and contributes to the literature, clinical practice and/or fills a gap(s) in knowledge	1
	3b	The Body must describe the main clinical findings, including symptoms and signs, if present	1
	3c	The Body must describe the main radiographic/histological/ laboratory/diagnostic findings	1
	3d	The Body must describe the main outcomes of treatment, if active treatment has been provided	1
	3e	The Conclusion(s) must contain the main "take-away" lesson(s), sometimes referred to as key learning point(s)	1
**Introduction**	4a	A background summary of the case(s) with relevant information must be provided	2
**Informed consent**	5a	A clear statement that informed, valid consent was obtained from the patient(s) must be provided	3
**Case report information**	6a	The age of the patient(s) must be provided	5-7
	6b	The gender of the patient(s) must be provided	5-7
	6c	The ethnicity of the patient(s) must be provided, if relevant	5-7
	6d	The main concern, chief complaint or symptoms of the patient(s), if any, must be provided	5-7
	6e	The medical history of the patient(s) must be provided, if relevant	5-7
	6f	The dental history of the patient(s) must be provided, if relevant	5-7
	6g	The family history of the patient if associated with the primary complaint must be provided, if relevant	Not applicable
	6h	The psychosocial history of the patient if associated with the primary complaint must be provided, if relevant	Not applicable
	6i	Genetic information, including details of relevant comorbidities and past interventions and their outcomes must be provided when possible, if relevant	Not applicable
	6j	Extra-oral findings must be provided, if relevant	Not applicable

	6k	General intra-oral findings must be provided when relevant, e.g. carious lesions, restorations, periodontal condition, soft tissues etc.	5-7
	6l	Important/relevant dates and times (in the text, or a table or figure) must be provided in chronological order	5-7
	6m	The diagnostic methods and the results for the specific tooth/teeth (e.g. pulp sensibility test, tenderness, mobility, periodontal probing depths, laboratory investigations, imaging techniques, or other special tests) must be provided	4-7
	6n	The diagnostic challenges, if any, must be provided	5-7
	6o	The diagnostic reasoning including other possible diagnoses that were considered must be provided	5-7
	6p	The active treatment (s) or intervention(s) performed, if any, must be provided	4-7
	6q	Any modifications to the proposed treatment(s) or intervention(s), if necessary, must be provided	Not applicable
	6r	The assessment method(s) used to determine the clinician-assessed and patient-assessed treatment outcomes and their results must be provided	4-7
	6s	Adverse and unanticipated events or consequences, if any, must be provided	Not applicable
**Discussion**	7a	The specific treatment(s) and intervention(s) (if any) must be discussed with reference to the relevant literature	8-10
	7b	The strengths of the case report and its importance must be discussed with reference to the relevant literature	8-10
	7c	The limitations of the case report must be discussed	9
	7d	The rationale for the conclusion(s) must be discussed	10
**Patient perspective**	8a	Feedback from the patient on the treatment and the care they received should be provided, if relevant	4
**Conclusion**	9a	Explicit conclusion(s), i.e. the main "take-away" lessons must be provided	10
	9b	Implications for clinical practice or future research must be provided	10
**Funding details**	10a	Sources of funding and other support (such as supply of instruments, equipment) as well as the role of funders must be acknowledged and described	10
**Conflict of interest**	11a	An explicit statement on conflicts of interest must be provided	10
**Quality of images**	12a	Details of the equipment, software and settings used to acquire the image(s) must be described in the text or legend	2
	12b	The reason why the image(s) was acquired and the rationale for its inclusion in the manuscript must be provided in the text	2
	12c	The circumstances (conditions) under which the image(s) were viewed and evaluated by the authors must be provided in the text	2
	12d	The resolution and any magnification of the image(s) or any modifications/enhancements (e.g. adjustments for brightness, colour balance, or magnification, image smoothing, staining etc.) that were carried out must be described in the text or legend	2
	12e	Patient(s) identifiers (names, patient numbers) must be removed to ensure they are anonymised	3

	12f	An interpretation of the findings (meaning and implications) from the image (s) must be provided in the text	5-7
	12g	The legend associated with each image must describe clearly what the subject is and what specific feature(s) it illustrates. Legends associated with images of patients must describe the age, gender and ethnicity of the person, if relevant	3
	12h	Markers/labels must be used to identify the key information in the image(s) and be defined in the legend or as a footnote	3
	12i	The legend of each image must include an explanation whether it is pre-treatment, intra-treatment or post-treatment and, if relevant, how images over time were standardised	3

***From: Nagendrababu V, Chong BS, McCabe P, Shah PK, Priya E, Jayaraman J, Pulikkotil SJ, Setzer FC, Sunde PT, Dummer PMH (2020) PRICE 2020 Guidelines for reporting case reports in Endodontics: A consensus-based development. *International Endodontic Journal* doi: 10.1111/iej.13285.**

For further details visit: http://pride-endodonticguidelines.org/price/
